# Kindchenschema and cuteness elicit interest in caring for and playing with young children, but less so when children are masked

**DOI:** 10.1038/s41598-022-15922-z

**Published:** 2022-07-13

**Authors:** Sabine Doebel, Nicole J. Stucke, Songhan Pang

**Affiliations:** Department of Psychology, George Mason University, Fairfax, VA USA

**Keywords:** Human behaviour, Psychology

## Abstract

Cuteness in the young has long been theorized to elicit care and protection. Most research on this topic has focused on human infants, despite theories suggesting that cuteness may elicit broader social interest that could support learning and development beyond infancy. In four experiments (*N* = 531 adults, 98 children), we tested whether ‘kindchenschema’—facial features associated with cuteness—and perceived cuteness elicit interest in playing with and caring for children, and whether masks disrupt these processes. Participants viewed images of children’s faces, masked or unmasked. Kindchenschema correlated with perceived cuteness and age, and these variables predicted adults’ interest in playing with and caring for children. Masks did not reduce cuteness ratings or interest in children, although they weakened relations between perceived cuteness and interest, and between perceived age and interest. Cuteness and related signals may guide adults’ interactions with children, fostering learning and development.

## Introduction

Cuteness is ubiquitous in the young—conveyed by physical characteristics, sounds, and even smells^1^—and has long been theorized to have evolved to motivate others’ care and protection^2^. Human infants display *kindchenschema* (e.g., larger heads, eyes, and chubby cheeks), which elicit perceptions of cuteness, prolonged attention, interest in caregiving, and reward activity in the brain^3–5^. These responses may be heightened in women of childbearing age^6^, but they are reliably elicited in parents and non-parents^7^, children and adults^8^, and males and females^9^. Responses have been elicited with explicit and implicit measures (e.g., looking time and implicit associations^9,10^), and have been found for non-human animals (e.g., kittens and puppies^8,11^) and even inanimate objects^12^. Adults even make positive judgments about baby-faced adults (e.g., that they are warm, trustworthy, and innocent^13,14^).

Accordingly, cuteness has been theorized to activate numerous psychological processes and behaviors beyond care and protection, including mentalizing, social engagement, empathy, and play^1,15^. In light of these ideas, one might expect cuteness to play an important role in guiding adults’ engagement with children. Adults rate younger children as more attractive and likable than older ones^16^, and they attribute more positive affect and helplessness to less mature child faces and voices^17,18^. However, downstream influences of kindchenschema and cuteness in children on interest and engagement with them have not been investigated.

In the reported research, we explore the possibility that cuteness in children affects others’ perceptions and interest in a way that could support their development. In four experiments (*N* = 629; 531 adults and 98 children) we used unmanipulated images of diverse children’s faces from the Child Affective Facial Expression (CAFE) stimulus set (age range: 2.75 to 7.5 years;^19^) to test whether kindchenschema, perceived cuteness, and perceived age are associated in children, and whether these variables predict interest in playing with and caring for children. We asked participants to rate the cuteness and age of the depicted children (Experiment 1) and to indicate their interest in playing with (Experiments 2–3) and caring for them (Experiment 4). We also obtained facial measurements to create 4 indices of kindchenschema that were appropriate for this diverse face stimuli set (adapted from^3,4^): face width/head length, forehead length/face length, eye width/face width, and head length/nose length. We combined these indices to create a single kindchenschema score for each image.

**Figure 1:**
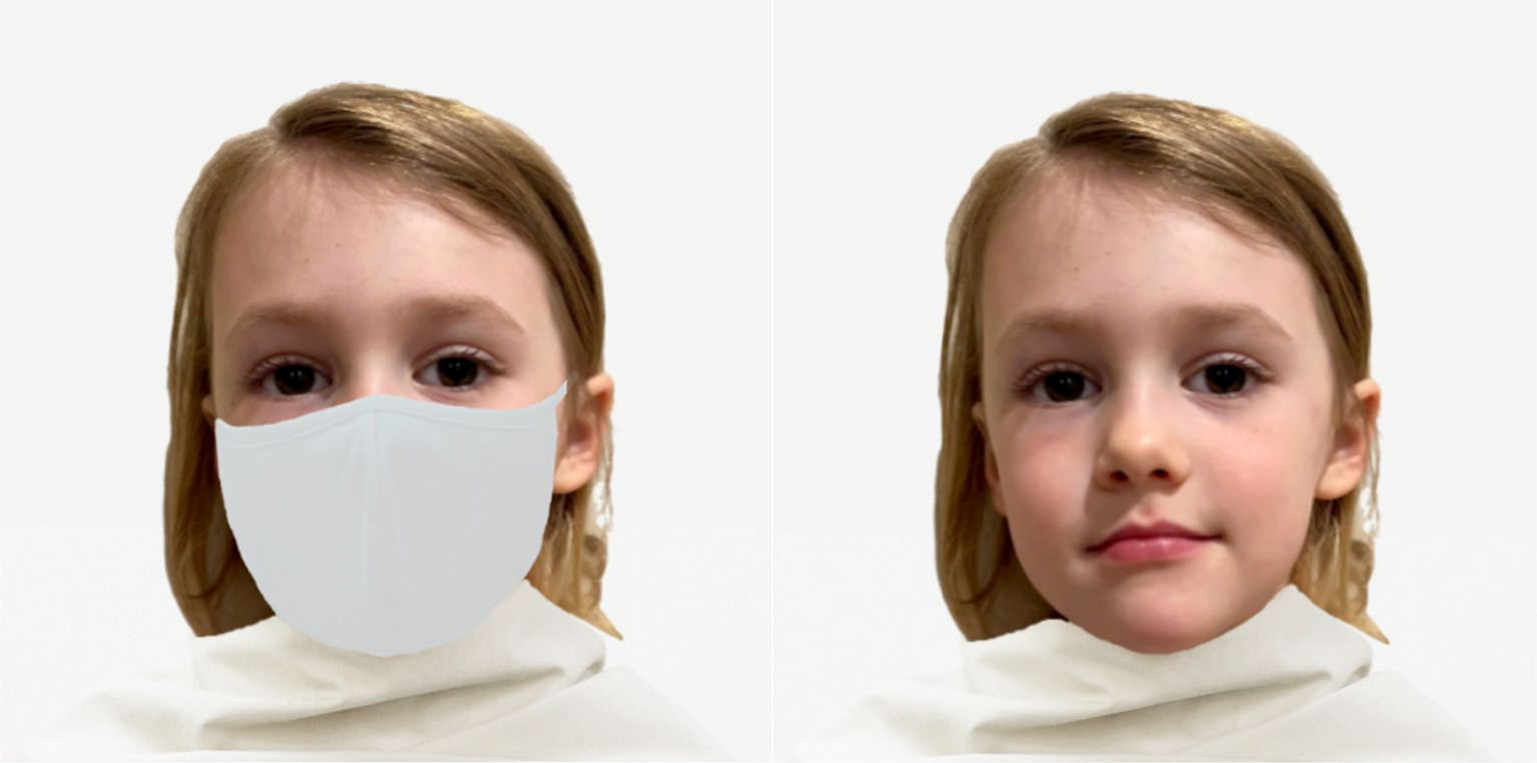
Example of face stimuli used in Experiments 1–4. Participants were randomly assigned to view faces that were masked (left) or unmasked (right).

Across experiments we also manipulated, between subjects, whether or not the faces were masked (Fig. 1), reasoning that if kindchenschema and cuteness foster interest in and concern for children, then obscuring some of this information could disrupt these processes. This question is particularly relevant given the global pandemic and the likelihood that many children will continue to wear masks for long periods of time for the foreseeable future. Gaining insight into how masks may affect others’ interest and engagement could prompt consideration of how to mitigate any negative consequences.

## Results

**Figure 2:**
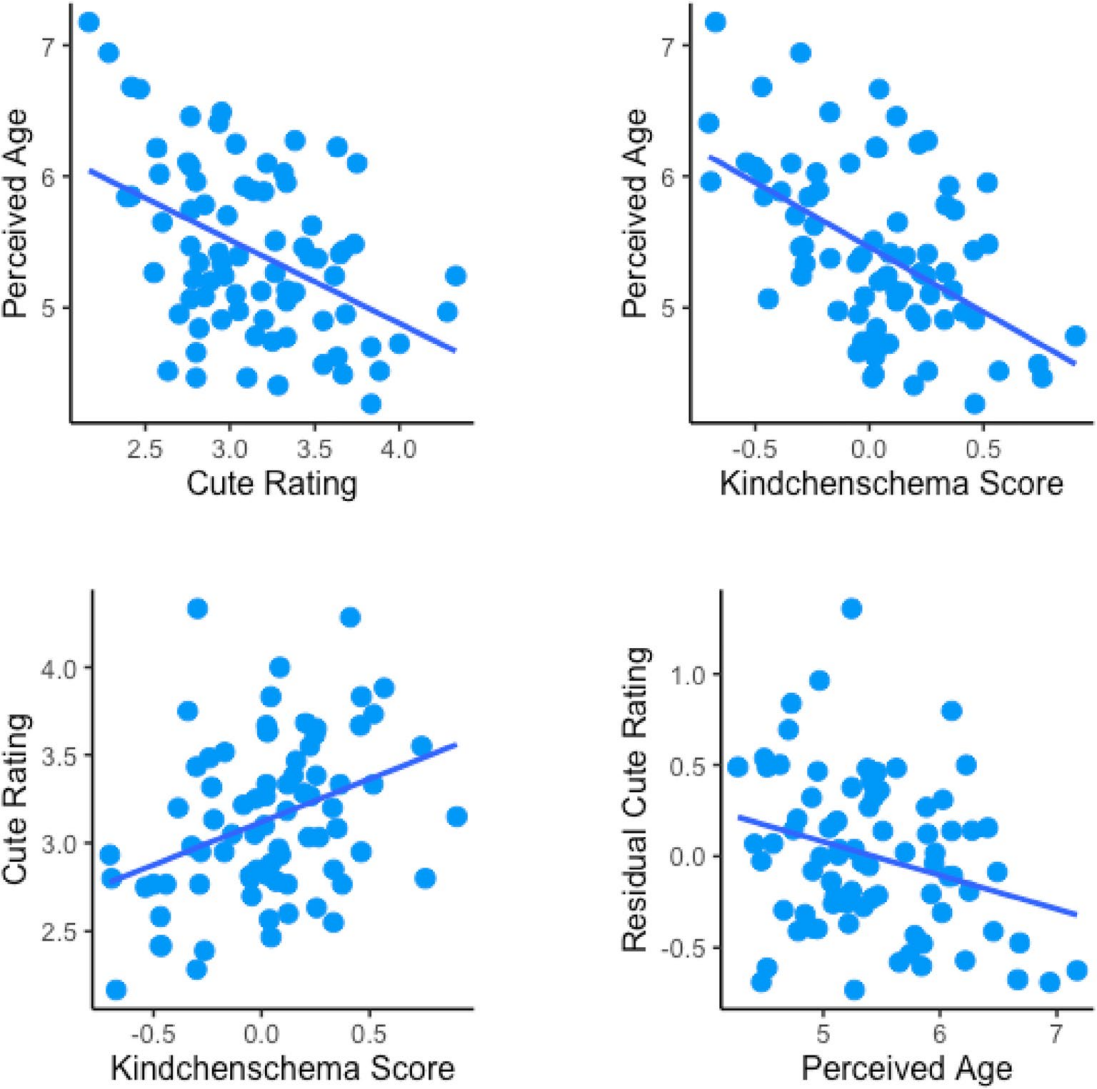
Results of Experiment 1. (*Top panel and Lower Left*) Perceived age, cute ratings, and kinchenschema scores for face images were correlated. (*Lower Right*) Cute ratings remained correlated with perceived age after partialling out variance related to kindchenschema scores. Plots show image means.

In Experiment 1 *(n* = 118 adults), we found that perceived cuteness and perceived age were related, $\chi$^2^(1) = 18.76, *p* < 0.001. Kindchenschema scores were also associated with perceived age, $\chi$^2^(1) = 14.68, *p* < 0.001, and with perceived cuteness, $\chi$^2^(1) = 11.57, *p* < 0.01 (Fig. 2). These results likely underestimate these relations because kindchenschema is conveyed by additional information not captured by our measurements (e.g., chubby cheeks, shape of nose, lips, eyes, and face). Consistent with this possibility, perceived age remained correlated with perceived cuteness after accounting for measured kindchenschema, $\chi$ ^2^(1) = 17.37, *p* < 0.001 (Fig. 2). We did not find the predicted main effect of masks on perceived cuteness, *p* > 0.5. Yet exploratory analyses indicated that masks weakened the associations between measured kindchenschema and perceived age, $\chi$^2^(1) = 74.05, *p* < 0.001, and between measured kindchenschema and perceived cuteness, $\chi$^2^(1) = 4.53, *p* < 0.05. This is not surprising given that the kindchenschema measurements involved landmarks that were not visible when children were masked, and thus could not be used to guide judgments. Yet it is important to note, as one might assume, that visible kindchenschema would provide comparable information. To further explore these results, we tested whether measurements of eye width over face width—the only kindchenschema index that was fully visible when faces were masked—predicted cuteness ratings, and found that it did not, *p* > 0.6. Conversely, a kindchenschema score comprised of the remaining facial indices (excluding the measurement of eye width over face width) did predict cuteness ratings for both masked and unmasked faces, $\chi$^2^(1) = 8.84, *p* < 0.01, but there was an interaction with mask condition, $\chi$^2^(1) = 7.87, *p* < 0.01, indicating the scores were more predictive when faces were unmasked.

What roles might kindchenschema and cuteness play in supporting interest in children? In Experiment 2, in a new sample of adult and child participants, we tested whether these variables might predict interest in playing with children, both on the part of adults and children themselves. Since prior research indicates that cuteness perception seems to emerge between 3 and 6 years of age^8,20^, we tested whether it would guide children’s play preferences and whether masks would reduce cuteness-based interest. We also tested whether masks, to the extent that they obscure some of children’s facial cuteness, disrupt interest in playing with children.

Adults’ interest in playing with the depicted children was predicted by kindchenschema, $\chi$^2^(1) = 5.22, *p* < 0.02, as well as the cuteness ratings obtained with a different sample in Experiment 1, $\chi$^2^(1) = 59.59, *p* < 0.001. The same pattern was found for perceived age ratings from Experiment 1, $\chi$^2^(1) = 15.16, *p* < 0.001; however, this relation was no longer significant after controlling for perceived cuteness (*p* > 0.38), consistent with perceived cuteness indexing age-related dependency and guiding interest.

We did not find the predicted main effect of masks on interest in playing with children; rather, adults reported *more* interest in playing with children wearing masks (*M* = 3.49) versus no masks (*M* = 3.22), $\chi$^2^(1) = 4.59, *p* < 0.05. As in Experiment 1, this may have been driven by a positivity bias for the partially occluded faces and/or by the neutral expressions of the unmasked faces.

Nevertheless, as predicted, masks affected the relation between cuteness ratings obtained in Experiment 1 and interest ratings obtained in the new sample, such that the relation was weaker when children were masked versus when they were not, $\chi$^2^(1) = 17.41, *p* < 0.001. The same pattern was found for perceived age ratings obtained in Experiment 1 and interest ratings obtained in a new sample, $\chi$^2^(1) = 5.72, *p* = 0.02.

In contrast to the findings with adult participants, child participants' interest in playing with the depicted children did not correlate with previously obtained cuteness ratings or measured kindchenschema, and there was no effect of masks, *p* > 0.05. Instead, children’s judgments were guided by several gender-related variables: the participant’s gender, the depicted child’s gender, and the match between the two. Specifically, female children were more interested in playing with depicted children, $\chi$^2^(1) = 10.50, *p* < 0.01, and children as a group tended to be more interested in playing with depicted females than with depicted males, $\chi$^2^(1) = 13.97, *p* < 0.001. Children also indicated more interest in playing with children of their own gender, $\chi$^2^(1) = 74.04, *p* < 0.001.

In Experiment 3 (*n* = 100 adults), we aimed to replicate these results and also test whether masks with different patterns would affect interest. We predicted that masks with positive or cute patterns might increase interest relative to plain masks or those with neutral patterns. If so, this could help attenuate any possible reduction in interest due to masks.

We replicated the findings from Experiment 2, with interest in playing being predicted by kindchenschema, $\chi$^2^(1) = 4.12, *p* < 0.05, and prior cuteness ratings, $\chi$^2^(1) = 52.28, *p* < 0.001. Masks weakened these relations, $\chi$^2^(1) = 5.84, *p* < 0.05, and $\chi$^2^(1) = 20.6, *p* < 0.001, respectively. Similarly, interest in playing was predicted by ratings of perceived age from Experiment 1, $\chi$^2^(1) = 18.16, *p* < 0.001, and this relation was weakened in the presence of masks, $\chi$^2^(1) = 3.58, *p* = 0.06. We found no evidence that masks or mask patterns influenced interest in playing, *p*s > 0.05.

It is possible that asking participants about their interest in playing with the depicted children led them to search for cues of readiness to play (e.g., smiles), and thus reported interest in playing with unmasked children may have been lower than it would have been otherwise, reducing the likelihood of detecting a main effect of masks. Thus, in Experiment 4 we repeated our procedure but instead asked about interest in caring for the depicted children, as in^3,4^.

As in our earlier experiments, we found no main effect of masks on interest in caring for children, *p* > 0.05. Interest in caring for children was predicted by kindchenschema, $\chi$ ^2^(1) = 4.18, *p* < 0.05, and prior cuteness ratings, $\chi$^2^(1) = 55.53, *p* < 0.001, and masks weakened these relations, $\chi$^2^(1) = 3.83, *p* = 0.05, and $\chi$^2^(1) = 21.84, *p* < 0.001. Similarly, interest in caring for children was predicted by ratings of perceived age from Experiment 1, $\chi$^2^(1) = 17.07, *p* < 0.001. This relation was also weakened in the presence of masks, $\chi$^2^(1) = 7.77, *p* < 0.01. Thus, whether participants are asked about interest in playing with or caring for children, we do not find evidence that masks affect their ratings; however, we do find consistent evidence that ratings are less guided by cues of age-related dependence—kindchenschema and perceived cuteness—in the presence of masks.

## Discussion

The reported research is the first to show that measured facial kindchenschema predicts perceived cuteness and interest in playing with and caring for children. These findings are consistent with prior theorizing regarding a broader role for cuteness beyond eliciting protection^1^ and calls for more research into how cuteness shapes adults’ interactions with children, and, by extension, children’s development. Here we focused on facial cuteness, however cuteness is multidimensional, extending to sounds and smells^1^ and possibly more (e.g., behaviors such as play and dancing). How might these signals encourage others not only to attend to young children but to interact with them in specific, nurturing ways? For example, adults modify their verbal input to babies and young children and this facilitates learning^21^. Cuteness may play a key role in eliciting and fine-tuning such verbal input to the infant or child.

Recent theories highlight that the extended childhood that humans enjoy may be an adaptation in its own right, fostering flexibility and extended learning^22^; cuteness in younger children may be one facilitating mechanism through which extended nurturing and learning occurs, by attracting others’ attention and interest, and allowing children freedom to explore. Future research could explore a broader age range of face stimuli to gain insight into how long children’s cuteness influences adults’ interest and how that interest changes over development.

Conversely, factors that might interfere with cuteness perception could disrupt these processes^1^. For example, depression affects emotion recognition and attribution^23,24^ as well as caregiver perceptions of and responsiveness to negative infant cues such as crying^25,26^, and thus might be expected to affect cuteness perception as well^1^. In short, there are many open questions about how kindchenschema and other cuteness signals may foster and fine-tune engagement with children that are deserving of exploration.

Previous studies have found that children notice and respond to cuteness as well^8^, so it is unlikely that this was unnoticed by children in our study; however, it did not factor into their reported interest in playing with the depicted children. The fact that children’s responses were predicted by three gender-related variables speaks against the possibility that our measure was insensitive or unreliable. The patterns are also consistent with prior findings that children of a certain age prefer same sex playmates^27^ and that girls tend to be more positively oriented to others than boys (e.g., they are more prosocial^28^). Thus, a plausible interpretation is that while children of this age can detect and respond to cuteness in humans, this information is not prioritized in the selection of playmates.

Contrary to our hypotheses, we did not find that masks reduce cuteness ratings or dampen overall interest in playing with and caring for young children. This is consistent with the possibility that other visible cues continue to encourage others’ interest and concern. Yet it is also possible that any negative effects of masks on interest may have been offset by lower reported interest in unmasked faces with neutral expressions. Participants may also have made optimistic inferences regarding the hidden parts of the face, such as that they are attractive, positive, or cute. This kind of positivity bias has been demonstrated previously, with higher attractiveness judgments for partially occluded versus unoccluded faces^29^.

Yet masks disrupted, to some extent, the perception of kindchenschema, as indicated by its weakened relations with perceived age and cuteness in the presence of masks. Interestingly, kindchenschema predicted perceived cuteness and age of masked faces, yet this was not driven, as one might expect, by visible measured kindchenschema (eye width over face width). While recent research suggests seeing the eyes may be more important than seeing other parts of the face in others’ judgments of cuteness and vulnerability^20,30^, our findings seem to suggest otherwise—eye size may convey less about cuteness and age than other features that are partially obscured by masks.

Cuteness ratings that were obtained in one sample for unmasked faces were less predictive of subsequent samples’ interest in playing with and caring for the depicted children when they were masked versus when they were not. The same pattern held between perceived age and interest in playing with and caring for depicted children. A possible explanation for these results is that participants’ judgments of masked faces are simply noisier as they are based on less information. Another possibility is that judgments of masked faces are influenced by what participants spontaneously imagine the faces looks like underneath the masks, which could be influenced by idiosyncratic experiences and representations of an average face^29^. Either way, the findings indicate that in the presence of masks, interest and concern are less influenced by the specific child’s kindchenschema and cuteness. Consideration of how to mitigate these effects when children are wearing masks for long periods of time may be warranted.

Because our kindchenschema measurements were obtained based on 2D images of faces, and the measurements themselves are somewhat crude indicators of age-related cuteness conveyed by children’s facial features, this research likely underestimated the influence of facial kindchenschema on interest. The subjective cuteness ratings offered a way around this limitation, as evidenced by cuteness and perceived age remaining associated after kindchenschema scores were partialled out. On the other hand, because masks obscure only part of the face and not other signals that are known to change with development (e.g., head, limb, and body proportions, voice), it is possible that other cues to age-related dependence can be relied upon in a somewhat compensatory way. For example, prior work suggests children’s immature thinking may influence adults' perceptions more than facial information^31^. Because our study used static images alone, in the absence of other cues, we do not know the extent to which masks affect others’ interest when other cues to youth are present.

Together, these findings suggest physical and potentially other manifestations of cuteness in children foster others’ interest and concern, potentially impacting learning and development. Research is needed to better understand these processes, and to learn more about the extent to which masks interfere with them.

## Method

The reported experiments were conducted with approval from the Institutional Research Ethics Board at George Mason University. All methods were performed in accordance with the relevant guidelines and regulations. All adult participants and parents of child participants provided informed consent to take part in the experiments, and child participants provided assent. Informed consent was obtained from the legal guardians of one child (who was not a participant in the study) for the publication of their image in an online open-access journal.

### Analytic approach across experiments

To conduct our analyses, we fit mixed-effects regression models of perceived cuteness and age, and interest in playing with and caring for children. These analyses were implemented using the *lmer()* function of the *lme4* package^32^ in R^33^. Random intercepts were included to account for item-level and subject-level variation. We used a model comparison approach^34^ to test whether a model with a predictor of interest (e.g., mask condition) better explained the data than a model without the predictor. The comparisons were implemented using Analysis of Variance, yielding Chi-square statistics and p-values. Analytic code and data for all experiments can be found on the Open Science Framework: https://osf.io/6mnsq/. Tables of our model estimates and statistical tests are provided in an online supplemental file.

### Experiment 1

#### Participants

118 participants (91 female, mean age = 23.77 years) were recruited and tested online via Qualtrics. Twenty-two participants were recruited from a database of families consenting to be contacted about research projects and 96 were recruited from a university subject participant pool. Fifty-eight participants identified as White, 12 as South Asian, 10 as Black/African American, 8 as East Asian, 3 as Native Hawaiian or Pacific Islander, 1 as Native American/Alaska Native, and 19 participants identified as multiracial or other. Nineteen participants identified as Hispanic or Latinx. Seven participants preferred not to respond. Data from an additional 10 participants were not included in the final dataset because they gave all but two or fewer images the same rating.

#### Face stimuli

The face stimuli were a subset of 79 chromatic images from the CAFE stimuli set^19^. The full CAFE stimuli set^19^ is available with permission via DataBrary. Permission was not available to reproduce or share these images in a publication, thus we obtained permission to photograph another child to illustrate the stimuli (Fig. 1). The images were of children’s faces (45 female, 34 male, age range = 33—89 months) wearing neutral expressions. The racial/ethnic breakdown of the images were as follows: 36 White, 14 Black/African American, 13 Latinx/non-White Hispanic, 9 Asian, and 7 South Asian children. We did not use windowing or other procedures that would focus attention exclusively on facial features because we aimed for a more naturalistic assessment and to avoid cueing participants to respond in terms of facial features.

#### Procedure

Following the completion of informed consent, participants were instructed: “In the following section, you will see a number of children's faces one at a time. For each child, we ask that you rate how cute you perceive the face to be and​​​​ provide an estimate of the child's age.”

Next, participants were show each image, sequentially, and were asked “How cute is this child?” and “How old is this child?” Cuteness ratings were provided as ‘stars’ on a 5-point scale with 1 to 5 stars as response options. Perceived age in years and months was entered by participants using a slider, with age in half year increments (e.g., 3.5 years; range 2.5–8 years).

We used perceived age rather than actual age in this and subsequent experiments because images did not accurately reflect objective sizes of children’s features (i.e., images were not cropped so as to reflect the objective size of the child’s face relative to other faces, and it was not possible to determine objective size). Thus, perceived age based on the perceived size of the head and features was considered a more reliable index.

Participants were randomly assigned to one of two conditions: a mask condition in which face images showed a plain surgical mask on the lower half of the face, covering the nose, and an unmasked condition in which the same faces were shown with neutral expressions and without a mask. The mask manipulation was between subjects in all of our experiments in order to minimize the likelihood that participants would infer the study goals and use the presence or absence of masks as their rating criterion.

We created the masked images superimposing a vector image of a real mask onto the faces of the depicted children from the CAFE stimulus set. The image of the mask was obtained from an uncopyrighted image of a child wearing the mask downloaded from https://unsplash.com. The background of the image as well as the child’s face was cropped from the image using a free, online tool (https://photoscissors.com). This procedure allowed us to make the image look realistic by adjusting the mask to fit the varying facial structures of each child (e.g., making the mask wider or longer to fit the child’s face; see Fig. 1 for an illustration).

Once participants completed their ratings, they clicked an arrow at the lower right-hand side of the screen to advance to the next image. Participants rated 80 images in total. Due to an error, participants rated two images of the same child taken on different occasions). All data related to the second image were removed from the dataset, leaving 79 images rated.

Faces were presented in one of two fixed orders that pseudorandomized the gender, race, and ethnicity of faces. Follow-up analyses with these variables included in models indicated no significant influence of these variables and no change in the pattern of results.

#### Kindchenschema measurement

One image could not be reliably measured because the head was tilted upward. Pictures had varying pixel dimensions. Deviating from^3,4^, we obtained kindchenschema measurements without manipulating head length (hl) to 500 pixels to reflect the raw dimensions that study participants observed. Following^3,4^, facial measurements using the unit of pixels were obtained by using the Photoshop ruler tool to measure distances between landmarks on the face. Circular landmarks were marked on the original images using the software Pixlr X (https://pixlr.com/x/) by aligning a coordinate axis on the face that connects the inner corner of the eyes and runs the midline of the face. Landmarks were placed on the following: vertex of the head, gnathion (bottom of the chin), outer edges of the face along the x-axis, endocanthi (inner corners of the eyes), exocanthi (outer corners of the eyes), nasion (nose base at the intersection of the x and y-axis), and subnasale (base of the nose below the tip). In measuring distances between landmarks, the endpoint of the Photoshop ruler tool was placed at the center of each landmark. The following distances were measured: face width (distances between outer edges of the face along the x-axis), forehead length (vertex to nasion), face length (nasion to gnathion), eye width (endocanthus to exocanthus), nose length (nasion to subnasale), and average eye width. Unlike^3,4^, we did not take measurements of nose width or mouth width, given our diverse face sample. Previous research utilized an all-Caucasian infant photoset, certain parameters deemed as "cute" (e.g., small mouth and nose widths) may apply less to diverse faces. A second rater measured a subset (*n* = 30) of the face images and the resulting ICC was 0.823, indicating good reliability.

### Experiment 2

#### Participants

Ninety-five adults (68 female, mean age = 25.51 years) and 98 children (44 female, mean age = 65 months; range = 4.0 to 6.99 years) participated. Of the adult sample, 30 were recruited from a database of families consenting to be contacted about research projects and 65 were recruited from a university subject participant pool. Forty-eight participants identified as White, 11 as East Asian, 10 as South Asian, 6 as Black/African American, 1 as Native Hawaiian or Pacific Islander, 1 as Native American/Alaska Native, and 15 participants identified as multiracial or other. Fifteen participants identified as Hispanic or Latinx. Three participants preferred not to respond. Data from an additional 20 participants were not included in the final dataset because they gave all but two or fewer images the same rating.

Of the child sample, 86 were recruited and tested online via Qualtrics and 12 were tested in-person in a child laboratory setting. Participants tested in-person proceeded through the experiment via Qualtrics in an identical fashion as they would have online. Sixty-five participants identified as White, 12 as East Asian, 5 as South Asian, 1 as Black/African American, and 15 participants identified as multiracial or other. Four participants identified as White Hispanic. Data from an additional 16 participants were not included in the final dataset because they gave all but two or fewer images the same rating.

#### Procedure

To minimize participant fatigue as these experiments were carried out online, we used cuteness ratings from Experiment 1 to select a subset of 30 images that were reliably rated but had a range of mean scores (range 2.17–4.34). We used these images for Experiments 2–4.

Following the completion of informed consent, participants were instructed: “You will be shown some pictures of kids from a local school. Some children you may feel you want to play and engage with. You will see one child at a time and will be asked how much you feel you would want to play with them.” For child participants, these instructions were read by a parent or experimenter. We chose this question because of its appropriateness for use with both children and adults, facilitating comparison. (In Experiment 4 we ask about interest in caring for the depicted children, following^3,4^). By obtaining cuteness and interest ratings in different experiments with different samples, we addressed limitations of previous research where participants may have been primed to respond to questions about interest in terms of perceived cuteness^3,4^.

Next, participants were shown each image, sequentially, and were asked “How much do you want to play with them?” Ratings were provided using a slider with the following mappings: 1 = “Really don't want to”, 2 = “Sort of don't want to”, 3 = “Not sure”, 4 = “Sort of want to”, 5 = “Really want to”. For child participants, parents were instructed to read these options to their children prior to requesting their verbal response.

### Experiment 3

#### Participants

218 participants (164 female, mean age = 21.40 years) were recruited and tested online via Qualtrics. Five were recruited from a database of families consenting to be contacted about research projects and 213 were recruited from a university subject participant pool. Ninety participants identified as White, 24 as Black/African American, 22 as South Asian, 20 as East Asian, and 55 participants identified as multiracial or other. Twenty-nine participants identified as Hispanic or Latinx. Seven participants preferred not to respond. Data from an additional 27 participants were not included in the final dataset because they gave all but two or fewer images the same rating.

#### Procedure

The procedure for this experiment was identical to that of Experiment 2. Participants were randomly assigned to see faces in of four conditions: faces without masks or faces with plain, positively-patterned, or neutrally-patterned masks. Positive patterns were designed based on prior findings suggesting that small, symmetrical, brightly-colored patterns involving rounded symmetrical shapes are perceived as positive (e.g., pink, yellow, and light blue swirls, stars, flowers, circles, and arches^35^). Neutral patterns were designed with neutral or muddy colors (beige, brown, grey) and larger and less symmetrical patterns and shapes. To add the patterns to the masks, we cropped images of each design to the shape of each mask, layered it over the images of the masked children, and altered the design transparency such that the design appeared to be embedded on the mask. Examples of these images are available in the online supplemental file.

### Experiment 4

#### Participants

100 participants (69 female, mean age = 26.72 years) were recruited and tested online via Qualtrics. Most participants (*n* = 59) were tested on-site. The remaining participants (*n* = 41) participated online. Participants tested in-person proceeded through the experiment via Qualtrics in an identical fashion as they would have online. Forty-one were recruited from a database of families consenting to be contacted about research projects and 59 were recruited from a university subject participant pool. Forty-five participants identified as White, 20 as East Asian, 13 as South Asian, 12 as Black/African American, 1 as Native American/Alaska Native, and 7 participants identified as multiracial or other. Fifteen participants identified as Hispanic or Latinx. Two participants preferred not to respond. Data from an additional 20 participants were not included in the final dataset because they gave all but two or fewer images the same rating.

#### Procedure

In this experiment participants were told that they would be presented with a set of 30 children's faces, shown on the screen one at a time. For each, we asked that they rate how much they would like to care for the pictured child. Participants were asked to rate each picture separately. For each image they were asked: “How much does the child make you feel that you would like to take care of them?” Ratings were given with a slider using a five-point Likert scale with the following mappings: 1 = “Would not very much like to take care of”, 2 = “Would not like to take care of”, 3 = “Not sure”, 4 = “Would like to take care of”, 5 = “Would very much like to take care of”.

## Supplementary Information

Supplementary Information.

## Data Availability

Preregistrations, deidentified data and code for the four experiments are deposited on OSF https://osf.io/6mnsq/. The CAFE stimuli set^19^ is available with permission via DataBrary.
