# Supplementary material for: Kindchenschema and cuteness elicit interest in caring for and playing with young children, but less so when children are masked
Source: Sci Rep. 2022 Jul 13;12:11903. doi: 10.1038/s41598-022-15922-z (PMC9279288; doi:10.1038/s41598-022-15922-z)
Supplement: Supplementary file 1 — Supplementary Information. [file 41598_2022_15922_MOESM1_ESM.docx]

**Supplemental File**

**S1. Model Estimates and Statistics for Experiments 1 to 4**

**Experiment 1**

*Estimates for Mixed Effects Model Predicting Perceived Cuteness from Condition*

|  | Model summary | | | Model comparison | | |
| --- | --- | --- | --- | --- | --- | --- |
|  | *β* | *SE* | *t* | *χ^2^* | *df* | *p* |
| *Fixed Effects* |  |  |  |  |  |  |
| Intercept | 3.18 | 0.09 | 34.27 |  |  |  |
| Condition | 0.09 | 0.16 | 0.54 | 0.29 | 1 | 0.59 |
|  | *s^2^* |  |  |  |  |  |
| *Random Effects* |  |  |  |  |  |  |
| Participant (intercept) | 0.78 |  |  |  |  |  |
| Item (intercept) | 0.15 |  |  |  |  |  |

*Estimates for Mixed Effects Model Predicting Perceived Age from Condition*

|  | Model summary | | | Model comparison | | |
| --- | --- | --- | --- | --- | --- | --- |
|  | *β* | *SE* | *t* | *χ^2^* | *df* | *p* |
| *Fixed Effects* |  |  |  |  |  |  |
| Intercept | 5.34 | 0.10 | 55.85 |  |  |  |
| Condition | -0.17 | 0.14 | -1.27 | 1.60 | 1 | .20 |
|  | *s^2^* |  |  |  |  |  |
| *Random Effects* |  |  |  |  |  |  |
| Participant (intercept) | 0.53 |  |  |  |  |  |
| Item (intercept) | 0.36 |  |  |  |  |  |

*Estimates for Mixed Effects Model Predicting Perceived Age from Kindchenschema, Condition, and Their Interaction*

|  | Model summary | | | Model comparison | | |
| --- | --- | --- | --- | --- | --- | --- |
|  | *β* | *SE* | *t* | *χ^2^* | *df* | *p* |
| *Fixed Effects* |  |  |  |  |  |  |
| Intercept | 5.37 | 0.09 | 58.62 |  |  |  |
| Kindchenschema Score | -0.74 | 0.18 | -4.00 |  |  |  |
| Condition | -0.19 | 0.14 | -1.39 |  |  |  |
| Kindchenschema Score x Condition | 0.50 | 0.06 | 8.62 | **74.05** | **1** | **<0.001** |
|  | *s^2^* |  |  |  |  |  |
| *Random Effects* |  |  |  |  |  |  |
| Participant (intercept) | 0.53 |  |  |  |  |  |
| Item (intercept) | 0.29 |  |  |  |  |  |

*Estimates for Mixed Effects Model Predicting Perceived Cuteness from Kindchenschema, Condition, and Their Interaction*

|  | Model summary | | | Model comparison | | |
| --- | --- | --- | --- | --- | --- | --- |
|  | *β* | *SE* | *t* | *χ^2^* | *df* | *p* |
| *Fixed Effects* |  |  |  |  |  |  |
| Intercept | 3.17 | 0.09 | 34.66 |  |  |  |
| Kindchenschema Score | 0.43 | 0.12 | 3.52 |  |  |  |
| Condition | 0.09 | 0.16 | 0.56 |  |  |  |
| Kindchenschema Score x Condition | -0.11 | 0.05 | -2.13 | **4.53** | **1** | **0.03** |
|  | *s^2^* |  |  |  |  |  |
| *Random Effects* |  |  |  |  |  |  |
| Participant (intercept) | 0.78 |  |  |  |  |  |
| Item (intercept) | 0.13 |  |  |  |  |  |

*Estimates for Mixed Effects Model Predicting Perceived Cuteness from Eye width/Face width Z-score*

|  | Model summary | | | Model comparison | | |
| --- | --- | --- | --- | --- | --- | --- |
|  | *β* | *SE* | *t* | *χ^2^* | *df* | *p* |
| *Fixed Effects* |  |  |  |  |  |  |
| Intercept | 3.17 | .09 | 34.68 |  |  |  |
| Eye width/face width score | .03 | .09 | 3.06 | .25 | 1 | .62 |
|  | *s^2^* |  |  |  |  |  |
| *Random Effects* |  |  |  |  |  |  |
| Participant (intercept) | 0.77 |  |  |  |  |  |
| Item (intercept) | 0.15 |  |  |  |  |  |

*Estimates for Mixed Effects Model Predicting Perceived Cuteness from Kindchenschema score Without Eye width/Face width, Condition, and Their Interaction*

|  | Model summary | | | Model comparison | | |
| --- | --- | --- | --- | --- | --- | --- |
|  | *β* | *SE* | *t* | *χ^2^* | *df* | *p* |
| *Fixed Effects* |  |  |  |  |  |  |
| Intercept | 3.18 | .09 | 34.71 |  |  |  |
| Kindchenschema Score (no Eye width/Face width) | .28 | .09 | 3.05 |  |  |  |
| Condition | .08 | .16 | .54 |  |  |  |
| Kindchenschema score x Condition | -0.1 | .04 | -2.8 | 7.87 | 1 | .005 |
|  | *s^2^* |  |  |  |  |  |
| *Random Effects* |  |  |  |  |  |  |
| Participant (intercept) | 0.78 |  |  |  |  |  |
| Item (intercept) | 0.13 |  |  |  |  |  |

**Experiment 2**

*Estimates for Mixed Effects Model Predicting Perceived Cuteness from Condition: Adult Participants*

|  | Model summary | | | Model comparison | | |
| --- | --- | --- | --- | --- | --- | --- |
|  | *β* | *SE* | *t* | *χ^2^* | *df* | *p* |
| *Fixed Effects* |  |  |  |  |  |  |
| Intercept | 3.35 | 0.10 | 34.89 |  |  |  |
| Condition | 0.27 | 0.12 | 2.17 | **4.59** | **1** | **0.03** |
|  | *s^2^* |  |  |  |  |  |
| *Random Effects* |  |  |  |  |  |  |
| Participant (intercept) | 0.33 |  |  |  |  |  |
| Item (intercept) | 0.17 |  |  |  |  |  |

*Estimates for Mixed Effects Model Predicting Perceived Cuteness from Condition: Child Participants*

|  | Model summary | | | Model comparison | | |
| --- | --- | --- | --- | --- | --- | --- |
|  | *β* | *SE* | *t* | *χ^2^* | *df* | *p* |
| *Fixed Effects* |  |  |  |  |  |  |
| Intercept | 3.29 | 0.10 | 33.98 |  |  |  |
| Condition | 0.03 | 0.17 | 0.20 | 0.04 | 1 | 0.84 |
|  | *s^2^* |  |  |  |  |  |
| *Random Effects* |  |  |  |  |  |  |
| Participant (intercept) | 0.66 |  |  |  |  |  |
| Item (intercept) | 0.07 |  |  |  |  |  |

*Estimates for Mixed Effects Model Predicting Interest in Playing from Prior Cuteness Ratings, Condition, and Their Interaction*

|  | Model summary | | | Model comparison | | |
| --- | --- | --- | --- | --- | --- | --- |
|  | *β* | *SE* | *t* | *χ^2^* | *df* | *p* |
| *Fixed Effects* |  |  |  |  |  |  |
| Intercept | 1.49 | 0.15 | 9.82 |  |  |  |
| Condition | 0.89 | 0.19 | 4.62 |  |  |  |
| Cute Mean | 0.60 | 0.04 | 13.64 |  |  |  |
| Condition x Cute Mean | -0.20 | 0.05 | -4.18 | **17.41** | **1** | **<0.001** |
|  | *s^2^* |  |  |  |  |  |
| *Random Effects* |  |  |  |  |  |  |
| Participant (intercept) | 0.33 |  |  |  |  |  |
| Item (intercept) | 0.02 |  |  |  |  |  |

*Estimates for Mixed Effects Model Predicting Interest in Playing from Prior Perceived Age Ratings, Condition, and Their Interaction*

|  | Model summary | | | Model comparison | | |
| --- | --- | --- | --- | --- | --- | --- |
|  | *β* | *SE* | *t* | *χ^2^* | *df* | *p* |
| *Fixed Effects* |  |  |  |  |  |  |
| Intercept | 5.33 | 0.46 | 11.63 |  |  |  |
| Condition | -0.30 | 0.26 | -1.12 |  |  |  |
| Perceived Age | -0.35 | 0.08 | -4.39 |  |  |  |
| Condition x Perceived Age | 0.01 | 0.04 | 2.39 | **5.72** | **1** | **0.02** |
|  | *s^2^* |  |  |  |  |  |
| *Random Effects* |  |  |  |  |  |  |
| Participant (intercept) | 0.33 |  |  |  |  |  |
| Item (intercept) | 0.10 |  |  |  |  |  |

**Experiment 3**

*Estimates for Mixed Effects Model Predicting Interest in Playing from Mask Condition (Plain Mask vs. No Mask)*

|  | Model summary | | | Model comparison | | |
| --- | --- | --- | --- | --- | --- | --- |
|  | *β* | *SE* | *t* | *χ^2^* | *df* | *p* |
| *Fixed Effects* |  |  |  |  |  |  |
| Intercept | 3.58 | 0.12 | 28.70 |  |  |  |
| Condition | -0.24 | 0.18 | -1.33 | 1.75 | 1 | 0.19 |
|  | *s^2^* |  |  |  |  |  |
| *Random Effects* |  |  |  |  |  |  |
| Participant (intercept) | 0.41 |  |  |  |  |  |
| Item (intercept) | 0.20 |  |  |  |  |  |

*Estimates for Mixed Effects Model Predicting Interest in Playing from Mask Condition (Positive Mask vs. Neutral)*

|  | Model summary | | | Model comparison | | |
| --- | --- | --- | --- | --- | --- | --- |
|  | *β* | *SE* | *t* | *χ^2^* | *df* | *p* |
| *Fixed Effects* |  |  |  |  |  |  |
| Intercept | 3.41 | 0.13 | 25.38 |  |  |  |
| Condition | 0.09 | 0.18 | 0.52 | 0.27 | 1 | 0.60 |
|  | *s^2^* |  |  |  |  |  |
| *Random Effects* |  |  |  |  |  |  |
| Participant (intercept) | 0.66 |  |  |  |  |  |
| Item (intercept) | 0.13 |  |  |  |  |  |

*Estimates for Mixed Effects Model Predicting Interest in Playing from Mask Condition (Positive Mask vs. Plain)*

|  | Model summary | | | Model comparison | | |
| --- | --- | --- | --- | --- | --- | --- |
|  | *β* | *SE* | *t* | *χ^2^* | *df* | *p* |
| *Fixed Effects* |  |  |  |  |  |  |
| Intercept | 3.58 | 0.13 | 26.79 |  |  |  |
| Condition | -0.08 | 0.18 | -0.46 | 0.21 | 1 | 0.65 |
|  | *s^2^* |  |  |  |  |  |
| *Random Effects* |  |  |  |  |  |  |
| Participant (intercept) | 0.59 |  |  |  |  |  |
| Item (intercept) | 0.16 |  |  |  |  |  |

**Experiment 4**

*Estimates for Mixed Effects Model Predicting Interest in Caring from Mask Condition*

|  | Model summary | | | Model comparison | | |
| --- | --- | --- | --- | --- | --- | --- |
|  | *β* | *SE* | *t* | *χ^2^* | *df* | *p* |
| *Fixed Effects* |  |  |  |  |  |  |
| Intercept | 3.61 | 0.09 | 38.71 |  |  |  |
| Condition | -0.08 | 0.10 | -0.86 | 0.74 | 1 | 0.39 |
|  | *s^2^* |  |  |  |  |  |
| *Random Effects* |  |  |  |  |  |  |
| Participant (intercept) | 0.21 |  |  |  |  |  |
| Item (intercept) | 0.11 |  |  |  |  |  |

*Estimates for Mixed Effects Model Predicting Interest in Caring from Prior Cuteness Ratings, Mask Condition, and Their Interaction*

|  | Model summary | | | Model comparison | | |
| --- | --- | --- | --- | --- | --- | --- |
|  | *β* | *SE* | *t* | *χ^2^* | *df* | *p* |
| *Fixed Effects* |  |  |  |  |  |  |
| Intercept | 2.41 | 0.16 | 14.79 |  |  |  |
| Cute Mean | 0.39 | 0.05 | 8.21 |  |  |  |
| Condition | -0.78 | 0.18 | -4.41 |  |  |  |
| Cute Mean x Condition | 0.23 | 0.05 | 4.68 | **21.84** | **1** | **<0.001** |
|  | *s^2^* |  |  |  |  |  |
| *Random Effects* |  |  |  |  |  |  |
| Participant (intercept) | 0.21 |  |  |  |  |  |
| Item (intercept) | 0.01 |  |  |  |  |  |

*Estimates for Mixed Effects Model Predicting Interest in Caring from Prior Perceived Age Ratings, Mask Condition, and Their Interaction*

|  | Model summary | | | Model comparison | | |
| --- | --- | --- | --- | --- | --- | --- |
|  | *β* | *SE* | *t* | *χ^2^* | *df* | *p* |
| *Fixed Effects* |  |  |  |  |  |  |
| Intercept | 5.02 | 0.39 | 12.72 |  |  |  |
| Perceived Age | -0.25 | 0.07 | -3.66 |  |  |  |
| Condition | 0.57 | 0.25 | 2.25 |  |  |  |
| Perceived Age x Condition | -0.11 | 0.04 | -2.79 | **7.77** | **1** | **0.01** |
|  | *s^2^* |  |  |  |  |  |
| *Random Effects* |  |  |  |  |  |  |
| Participant (intercept) | 0.21 |  |  |  |  |  |
| Item (intercept) | 0.06 |  |  |  |  |  |

**S2. Supplemental Figures**


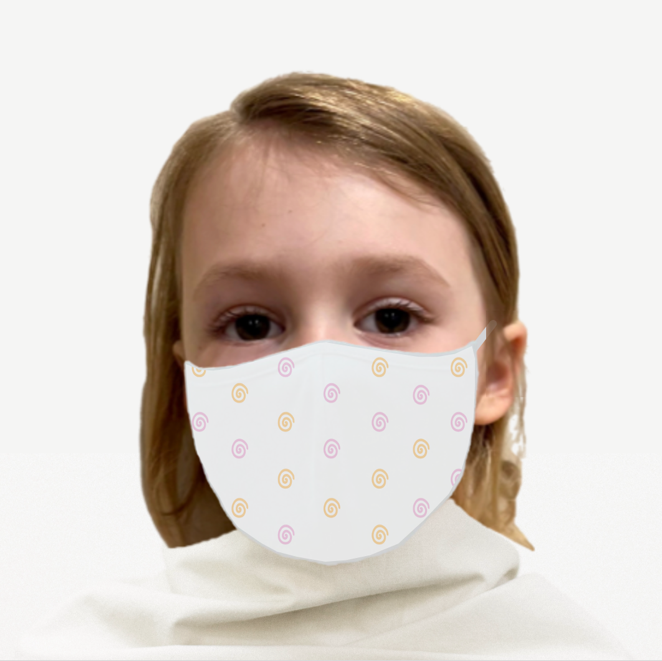

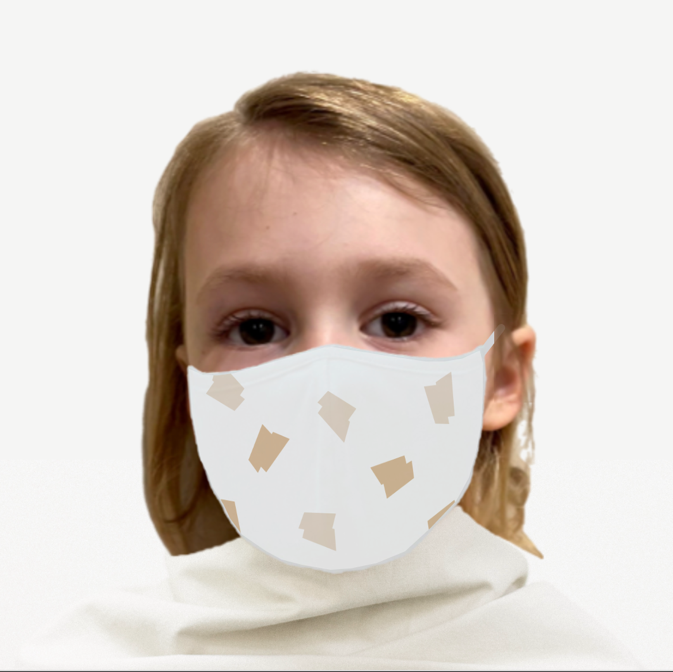


***Fig. 3.*** Example of face stimuli used in Experiment 3. Participants randomly assigned to one of four conditions in which they were shown child faces that were unmasked or wearing plain masks (as in Experiment 2, illustrated in Figure 1), masks with positive patterns (left) or masks with neutral patterns (right).
